# Preoperative Assessment of Adult Patients for Intracranial Surgery

**DOI:** 10.1155/2010/241307

**Published:** 2010-03-31

**Authors:** Vanitha Sivanaser, Pirjo Manninen

**Affiliations:** Department of Anesthesia, Toronto Western Hospital, University Health Network, University of Toronto, Toronto, ON, Canada M5T 2S8

## Abstract

The preoperative assessment of the patient for neurosurgical and endovascular procedures involves the understanding of the neurological disease and its systemic presentation, and the requirements of the procedure. There is a wide spectrum of different neurosurgical disorders and procedures. This article provides an overview of the preoperative evaluation of these patients with respect to general principles of neuroanesthesia, and considerations for specific intracranial and vascular neurosurgical and interventional neuroradiological procedures.

## 1. Introduction

The preoperative assessment of any patient is an integral part of safe anesthesia practice. Neurosurgery covers a wide spectrum of well-established procedures, but there are also areas that are rapidity expanding such as functional neurosurgery, awake craniotomy and interventional neuroradiology. The purpose of the preoperative assessment is for the anesthesiologist to review the patient's medical history including the neurological events, to obtain an understanding of the disease and its systemic manifestations, to ensure optimization of all comorbidities, and to prepare and plan for an appropriate and safe anesthetic. This article will cover the general preoperative approach for neurosurgical patients as well as considerations for specific intracranial and vascular neurosurgical and interventional neuroradiology procedures.

### 1.1. Preoperative Assessment

Neurosurgical patients vary widely in their presentation, from an alert and coherent patient presenting for an elective procedure to a patient with depressed neurological status due a devastating neurological insult for an emergent procedure. Hence, the diagnosis, preoperative physical and detailed neurological status, and the urgency of the procedure are important preoperative considerations to formulate an appropriate anesthetic plan [1]. The time and place of the preoperative interview will vary; for an elective procedure this may be in an anesthesia preoperative consult clinic several weeks before surgery, for emergency procedures in the neurosurgical critical care unit. In patients with a decreased level of consciousness, obtaining of history and pertinent information may be challenging and may only be obtainable from family members, caregivers, or medical notes. Previous surgical procedures and anesthetic notes offer valuable information and should be included. 

In addition to the routine assessment and preparation of any preoperative patient, emphasis should be placed on the review of the neurological system and comorbidities of the disease and of the patient. A neurological history is mandatory and should include the type and location of the lesion, symptoms, and medications related to the neurological problem, as well as the plan for treatment which may involve both surgery and/or endovascular therapy. Questions that will help illicit further information include a history of seizures, neurological deficits, signs and symptoms of raised intracranial pressure (ICP) such as headaches, nausea, vomiting, confusion, and a history of transient ischemic attacks (TIA) or stroke. Neurological examination should include the level of consciousness and the neurological physical examination which involves the status of the sensory and motor systems and evaluation of cranial nerves. 

Preoperative cardiovascular disturbances are common in patients undergoing neurosurgical interventions and include blood pressure fluctuations, electrocardiographic abnormalities, arrhythmias, and myocardial ischemia or failure. They can occur as consequences of central neurogenic effects on the myocardium and the autonomic nervous system or concurrently associated medical conditions. Preexisting cardiac disease should be identified, both symptomatic and asymptomatic. The decision to perform further diagnostic evaluations should follow established guidelines [2]. Multiple studies have reported improved outcome in patients receiving perioperative beta blockers, however newer studies have reported that perioperative beta blockers may not be effective if heart rate is not well controlled or in low risk patients [3, 4]. In a recent retrospective review of patients for noncardiac surgery, acute surgical anemia with a reduction in hemoglobin greater than 35% from baseline increased risks of cardiac complications in beta-blocked patients hence suggesting that the transfusion triggers should be higher for elective surgical patients on beta blockers [5]. The POISE trial showed a significant reduction in the primary outcome of cardiovascular events and a 30% reduction in myocardial infarction rates, but a significant increase in 30 day stroke and mortality [6]. The current ACC/AHA Guidelines on the perioperative beta blocker administration advocates that perioperative beta blockade should be used in patients on beta blockers and those with positive stress test undergoing major vascular surgery [7]. It also stresses that acute administration of beta blockers without titration may be harmful. Statins have been shown to improve perioperative cardiac outcome, hence are continued in patients currently taking them [7]. 

Review and optimizing of the respiratory system is important to ensure adequate oxygenation and ventilation intra and postoperatively. Patients with acute and chronic pulmonary disease need to have their disease stabilized preoperatively. Patients with neurological disorders may have respiratory complications such as aspiration of gastric contents and pneumonia, which can adversely affect neurologic outcome and survival. Neurogenic pulmonary edema may occur in patients with brain injury, subarachnoid hemorrhage (SAH), and stroke. History of smoking and cessation of smoking should be part of the preoperative evaluation [8], though most studies have not been able to identify preoperative smoking as an independent risk factor for major cardiovascular events [9]. Smoking diminishes the measures of cardiovascular function such as maximal exercise capacity and endothelium mediated vasodilatation, thus it is plausible that even brief period of cessation may be of benefit. Cessation of cigarette smoking should ideally begin at least 6–8 weeks prior to surgery as this period of abstinence is associated with improved pulmonary function and overall perioperative morbidity [10, 11]. History of obstructive sleep apnea (OSA) may influence the intra and postoperative care of the patient. There are specific groups of patients, such as in acromegaly and Cushing's disease, where there may be a high incidence of OSA. Clinical symptoms include daytime somnolence excessive snoring and fragmented night sleep. The STOP questionnaire is a validated screening tool for OSA [12]. Additional information on the body mass index, age, neck circumference, and gender should also be sought to increase the detection of the OSA patients [13]. The incidence of perioperative adverse events is increased in OSA patients [12]. 

Other medical disorders such as diabetes, renal impairment, and hepatic disease will also affect the anesthetic management of the patient and need to be optimized. There is an adverse association of hyperglycemia and neurosurgical outcome [14]. In a prospective analysis, the initiation of tight glycemic control reduced the mortality in neurosurgical patients and other critically ill patients [15]. In patients undergoing image-guided stereotactic brain biopsy, blood glucose levels less than 200 mg/dL was associated with almost a 100% positive predictive value of morbidity [16]. In general, blood sugar should be monitored frequently with the goal of avoidance of either hypo or hyperglycemia, and the maintenance of sugar levels between 100 mg/dL and 150 mg/dL. 

In the preoperative period neurosurgical patients often present with rapid changes in intravascular volume precipitated by hemorrhage, dehydration, diuretics and mannitol administration, and fluid restriction. Fluid management in the neurosurgical patient takes into consideration the restoration of intravascular volume, the maintenance of cerebral perfusion pressure and the avoidance of hyperglycemic and hypotonic fluids [17]. Iso-osmolar solutions, such as plasmalyte and 0.9% saline are used as they do not change the plasma osmolality, and therefore do not increase brain water content. Glucose-containing solutions and hypo-osmolar solutions, such as lactated Ringer's are avoided as plasma osmolality is reduced, consequently brain water content increases, worsening cerebral edema [18]. The administrations of medications, such as mannitol, will need to be carefully titrated in the presence of renal impairment and congestive cardiac failure.

### 1.2. Preoperative Medications

All current ongoing medications and allergies need to be reviewed. The preoperative continuation of most ongoing medications is usually straightforward but continuing of medications specific for neurological disorders will depend on recommendations from the neurosurgeon or neurologist. Patients on antiepileptic medications are known to have adverse effects from these medications as well as intraoperative pharmacokinetic interactions. Patients who have been placed on preoperative dexamethasone may present with elevated blood glucose levels which require careful monitoring [19, 20]. Patients with cerebral insufficiency may be on antiplatelet agents or anticoagulants for treatment of acute stroke, or secondary prevention of strokes from other disease as with patients with cardiac disease including coronary artery stents, prostatic heart valve, and intracardiac thrombus. The decision to stop this therapy before surgery is controversial and the planning should ideally be clarified during the preoperative assessment [21, 22]. Consultation with specialists such as hematologist, cardiologist and the surgeon is useful. The risks and the benefits of discontinuing or continuing therapy should also be discussed with the patient and family. Factors to consider are the urgency of the procedure and the presence of thrombotic or hemorrhagic risks. Recommendations for elective surgery with high hemorrhagic risk, such as intracranial and spinal procedures and moderate to high thrombotic risks suggest that aspirin should be continued, but to withhold clopidogrel and if the neurological and cardiovascular risks are low, the antiplatelet agents should be withdrawn (7 days for aspirin, 10 days for clopidogrel and 14 days for ticlopidine) [22, 23]. Further consultation is needed when major thrombotic factors exist such as when the time interval from bare metal stent insertion is less than six weeks or less than 12 months for patients with drug eluting stents. Patients who are on anticoagulation with agents such as warfarin need further consideration to determine the time interval to stop its administration (4-5 days preoperatively) and if anticoagulation with low molecular weight heparin and/or unfractionated heparin is needed in the interval [21]. Additional preoperative coagulation profile investigations may be needed. Latex allergy has to be excluded from patients who have had repetitive surgical procedures preformed, such as for spinal bifida. Likewise an allergy to contrast medium or protamine sulfate will have important considerations in the patient undergoing radiological and endovascular procedures. There are subgroups of disorders, where patients may be on chronic pain medication necessitating the planning for perioperative pain management. 

The presence of preoperative anxiety is high among neurosurgical patients. A recent study found that there was an increased need for information, especially about surgery, in patients with high levels of preoperative anxiety [24]. Preoperative treatment with anxiolysis or opioids is a controversial subject [25]. Patients who have increased ICP, or are obtunded are at a high risk of developing respiratory depression and even further increases in ICP with any sedation. Generally, for most neurosurgical patients with intracranial disease, if any preoperative sedation is to be used, it should be administered to the patient in a monitored environment where its reversal and airway management can be easy performed. 

### 1.3. Physical Examination

Physical examination of the patient needs to emphasize the neurological, as well as the cardiac and respiratory systems. Documentation of the assessment of neurological system is helpful for continued patient care. This includes patient's preoperative cognitive function (level of consciousness, communication, and intellect), and language (ability to communicate in written and verbal form). The Glasgow Coma Scale (GCS) is a standard means of assessment of the neurological state (Table 1) [26]. The GCS can assist the anesthesiologist in patient evaluation and the urgency of the need of intubation and neurosurgical intervention. A brief examination of the sensory and motor function is needed with documentation of any deficits, such as a weakness or loss of sensation of the extremities. Cranial nerve involvement or dysfunction can affect patient management during anesthesia. Occulomotor nerve controls the pupillary size and response to light. Preoperative pupil size should be documented for subsequent assessment of anesthetic depth and for comparison with postoperative pupil changes. The facial nerve can be damaged by face mask ventilation, surgery, and positioning. A baseline evaluation and documentation of any abnormality is appropriate prior to induction of anesthesia. Stimulation of the glossopharyngeal nerve by an oral airway or laryngoscope results in severe pain that radiates to the jaw and the ear. These patients can experience reflex bradycardia and hypotension during painful episodes secondary to the afferent discharge of the glossopharyngeal nerve. The vagus nerve supplies motor fibers to the pharynx, palate, larynx and the trachea. Hoarseness of the voice may indicate damage to the vocal cords. 

The establishment of a preoperative blood pressure level is helpful for intra- and postoperative decisions for appropriate blood pressure management [27, 28]. The presence of cardiac abnormalities such heart murmurs, abnormal heart rate and rhythm, poor peripheral pulses, signs of cardiac failure and the presence of carotid bruit will require further investigations and treatment. Respiratory system examination involves the assessment of pulmonary function and of the airway. Any signs of new respiratory disease should be investigated and treated. 

A thorough examination of the airway must be completed and documented using standard assessment guidelines [29]. The upper lip bite test has been shown to have a high accuracy and specificity, when compared with the thyomental distance, the inter incisor gap and the sternomental distance and may be useful in patients with restricted mouth opening and limited neck mobility [30]. Specific pathophysiological alterations, such as the presence of raised ICP, SAH, acromegaly, and cervical spine disease, will influence the technique chosen for securing of the airway. The presence of a difficult airway may further add risk to intubation in these patients as hypoxemia and hypercarbia exacerbate secondary neurological injury. Preoperative planning and discussion is required to ensure that the safest technique(s) is chosen. Fibreoptic intubation is not uncommon in neurosurgery, with 17% documentation in a review of 1612 cases [31]. While there are multiple factors that contribute to a difficult airway, in neurosurgery it is frequently seen in hypophyseal, craniofacial, and chronic spine pathology.

### 1.4. Laboratory Investigations

The ASA practice advisory for preanesthesia evaluation supports that routine preoperative investigations should not ordered, but rather that the tests should be selected based on type of procedure and the accompanying co morbidities, for the purpose of guiding and optimizing the preoperative management [32]. The test results are usually acceptable for up to six months prior to surgery provided the patient's medical history has not changed significantly. Blood loss as a result of surgery is common; one needs to ensure that there is an appropriate cross match for blood transfusion for each procedure. Severe anemia risks tissue hypoxia from impaired oxygen delivery. In neurosurgical patients, hematocrit (Hct) of 30–33% ensures optimal combination of oxygen carrying capacity and viscosity [17]. Base line hemoglobin (Hb) is shown to predict the need for subsequent transfusion in patients undergoing surgical procedures associated with significant blood loss [33], hence can be used to aide in planning perioperative transfusion. Theoretically coagulation tests may detect a predisposition to perioperative hemorrhage, however investigations of these tests show a low yield of discovering unsuspected disease, and is not predictive of perioperative hemorrhage if abnormal [34]. The preoperative assessment is not complete without the review of neuroradiological images such as the computer tomography (CT), magnetic resonance imaging (MRI), and angiography. Valuable information pertaining to the size of the lesion, its location, possible vascularity, and the surrounding structures can be obtained. 

### 1.5. Communication

Preoperative assessment also includes a thorough discussion with the patient and family of the techniques of anesthesia, requirements for the specific procedure such as invasive monitoring, the risks associated with anesthesia and with the patient's medical comorbidities, and the plans for the postoperative care, such as postoperative ventilation and pain management. Some procedures are performed as day surgery and the assessment of the ability of the patient and family to do this, plus instructions need to be clarified during their preoperative interview [35]. Appropriate instructions are given with respect to preoperative medications and fasting. The actual procedure for obtaining consent for anesthesia/surgery varies amongst institutions. This can be difficult and challenging in neurosurgical patients, as some will have depressed levels of consciousness, or diminished capacity to comprehend. Appropriate institutional procedures need to be followed. Finally, but perhaps the most important aspect of preoperative preparation is the communication with the surgeon and/or neuroradiologist. Pertinent information such as the urgency and possible difficulties of the procedure, positioning, use of intraoperative neurologic monitoring and the plan for postoperative care helps in the preparation of the patient.

## 2. Supratentorial Intracranial Tumors

The presentation of the patient with a supratentorial tumor will vary depending on the type of tumor, its location and size, and proximity to vital structures. The documentation of any altered level of consciousness, signs, and symptoms of raised ICP, sensory or motor deficits and seizures is essential. Tumors operated upon are usually meningiomas, gliomas, ventricular colloidal cysts or basal cistern epidermoids. Meningiomas can be technically demanding operation and is often associated with significant blood loss, while gliomas often undergo debulking with little risk of bleeding. It is also useful to review the CT and MRI imaging to ascertain characteristics of the tumor such as vascularity, size, location, and its proximity to a sinus and presence of increased ICP. Other aspects to evaluate are the hydration and volume status of the patient, especially those scheduled for emergency surgery. This can be assessed in terms of fluid intake, history of nausea and vomiting, diuresis with mannitol therapy for increased ICP, and the presence of inappropriate secretion of anti diuretic hormone syndrome (SIADH), or cerebral salt wasting. Preoperative management of the electrolyte imbalances may be needed. For patients who receive preoperative steroids, dexamethasone, and antiepileptic such as phenytoin or fosphenytoin, the drugs should be continued preoperatively. Dexamethasone will reduce tumor cerebral edema and this often leads to substantial symptomatic relief [36]. However, its use is associated with an increase in blood glucose levels [19, 20]. The administration of premedication for sedation can be risking in this group of patients. If respiratory depression results with increasing hypercarbia, there may a marked elevation in ICP in patients who have been well compensated when fully awake. 

## 3. Awake Craniotomy for Tumor Surgery

Awake craniotomy for tumor surgery is often used when the lesion is adjacent to eloquent cortex, such as speech or motor function [37]. Benefits of awake craniotomy include better preservation of eloquent function; shorter hospitalization and therefore reduction in cost [38]. These procedures require careful patient selection as part of preoperative preparation. Patients with anxiety disorders, claustrophobia, low tolerance to pain, and psychiatric disorders are not always suitable candidates for this procedure as patient cooperation and understanding are factors that ensure success to the procedure. The degree of anxiousness, tolerance to pain and the ability to cooperate are factors to be noted during the preoperative assessment. Choosing an awake technique depends on several other factors other than patient's physiological profile, such as location of the lesion, the presence of obstructive sleep apnea, seizures and nausea and vomiting [39]. Based on the clinical symptoms, signs, and imaging, evaluation of the degree of intracranial hypertension is documented. The hemorrhagic risk of the procedure is determined depending on the type of lesion and procedure. There are different techniques of awake craniotomy, such as asleep-awake-asleep or monitored anesthesia care with conscious sedation. With each technique, airway assessment is of extreme importance, predictors of difficult airway and factors that can favor upper airway obstruction such as obesity and OSA should be noted as the anesthesiologist must plan for possible emergent manipulation of the airway in a difficult position [29]. Preparation of the patient includes psychological preparation, reassurance and educating the patient regarding the events of the procedure including various tests during mapping.

## 4. Pituitary Tumor

Tumors of the pituitary gland are heterogeneous. Anesthetic care requires an understanding of the complex pathophysiology secondary to the patient's dysfunction of their endocrine system [40]. Pituitary tumors are classified according to size at time of diagnosis: microadenomas are less than 1 cm in diameter, macroadenomas are greater than 1 cm and their function: secreting, nonsecreting. Patient's symptoms will vary depending on the type of hormones secreted, the size and possible mass effect of the tumor (Table 2). Nonfunctioning tumors are more likely to be macroadenomas and to present with compressive symptoms such as headaches and visual changes. Medical evaluation is undertaken to optimize comorbidities associated with the specific endocrine dysfunction. Patients with acromegaly and Cushing's disease have unique systemic manifestations [40]. With acromegaly, there are multiple changes in the patients' physical structure which will impact on anesthesia, such as the large body mass as well as an unpredictable and difficult airway and OSA [41]. Detailed evaluation of the cardiac system is needed, as this may be a cause of morbidity and mortality [41, 42]. Patients with Cushing's disease present with multiple medical issues such as hypertension, glucose intolerance, myelopathy, osteoporosis, obesity, and OSA. Features such as obesity, moon facieses, buffalo hump and OSA may be associated with a difficult airway [43, 44]. Additional laboratory investigations are directed towards detecting endocrine and electrolyte abnormalities. Sodium imbalances may indicate posterior pituitary dysfunction in the form of diabetes insipidus or SIADH (Table 3). All endocrine related medications need to be continued. Patients with preoperative hypopituitarism should receive steroid and hormone replacement therapy perioperatively [45, 46]. Drug administration and therapy is guided by laboratory investigations and is often overseen by an endocrinologist.

## 5. Posterior Fossa Surgery

Posterior fossa surgery provides unique concerns and problems for the anesthesiologist. Patients may present various symptoms such as dysphagia, loss of gag reflex, laryngeal nerve dysfunction, and altered states of consciousness. Preoperative documentation of these is important for assessment of the neurological status at emergence and for planning of postoperative care. Patients with a diminished gag reflex may require continued intubation postoperatively. Positioning is more complicated and may be park bench, prone or sitting. In the lateral and prone position patients are at increased risk for peripheral nerve injury, eye injury and postoperative blindness [47]. Review of possible risk factors for these complications should be considered. In the sitting position there are increased risks of quadriplegia, paraplegia, facial edema, macroglossia, and venous air embolism (VAE). The incidence of VAE is high (39%) in the sitting position [48]. Preoperatively one needs to rule out the presence of an intracardiac (persistent foramen ovale) or an intrapulmonary shunt [49]. Transesophageal echocardiography and transcranial Doppler ultrasound are used to assess for a persistent foramen ovale [50, 51]. The presence of a persistent foramen ovale is an absolute contraindication to the sitting position. Relative contraindications include atherosclerotic cardiovascular disease, hemodynamic instability, and severe cervical canal stenosis. Patients with chronic pain from cranial nerve disorders such as trigeminal neuralgia present for procedures such as rhizotomy and injection of glycerol or microvascular decompression. The major preoperative consideration of this group of patients is their use of chronic pain medication.

## 6. Cerebral Aneurysm

The extent of the preoperative assessment of a patient with a cerebral aneurysm will be determined by their clinical presentation. Patients with intact aneurysms may be completely asymptomatic, in contrast to the patient with an acute SAH [52]. The neurological assessment of the patient with SAH should include the a grading such as World Federation of Neurosurgical Surgeons and GCS, and location, size, and number of aneurysms. Many multisystem manifestations may occur after a SAH. Intracranial complications include rebleeding, vasospasm, hydrocephalus, clot, and seizures. Cardiac complications include abnormalities in rhythm and electrocardiographic changes, elevated cardiac enzymes, and myocardial dysfunction [53–55]. Neurogenic pulmonary edema can occur at time of cerebral insult or up to two weeks after SAH [56]. There may be controversy as to best patient management when cardiac abnormalities are present, that is, whether to delay surgery for cardiac investigations. The decision to proceed with surgery needs to be balanced with the risk of rebleeding and needs to be discussed with the surgeon or radiologist. Electrolyte abnormalities including hyponatremia, hypokalemia, hypomagnesaemia and hypocalcaemia may be encountered and will need preoperative correction. Review of radiological imaging helps to determine the extent of the bleed, size and location of the aneurysm. Some patients present with cerebral vasospasm and are treated with “triple H therapy” (induced hypertension, hypervolemia, and hemodilution). Hypervolemia, especially in patients with a poor cardiac reserve, can result in pulmonary edema. Most patients are started on a calcium blocker, usually nimodipine, and this should be continued preoperatively. Communication is essential with the surgeon regarding patient positioning, likelihood of temporary clip application, and need for hemodynamic manipulations and cerebral protection.

## 7. Cerebral Arteriovenous Malformation

Patients with an AVM present with an SAH, neurological deficits, seizures, headaches or may be completely asymptomatic. A cerebral aneurysm may also be associated with the AVM. Many of the anesthetic issues involved in treatment of cerebral aneurysms are also applicable to the AVM group. The important consideration in surgical AVM resection is attentiveness to perioperative blood pressure management. Induced hypotension is frequently used intraoperatively to reduce bleeding and postoperatively strict control of the blood pressure is often needed to minimize complications such as bleeding and hyperperfusion syndrome [57]. Availability of blood for transfusion is essential, as blood loss can be substantial during the surgical resection.

## 8. Carotid Artery Stenosis

Patients scheduled for a carotid endarterectomy can present with TIA, completed stroke or may be asymptomatic. Common symptoms include changes in vision (amaurosis fugax), speech, or weakness of the extremities. Physical examination may reveal a high pitch bruit at the origin of the internal carotid artery, changes in the retina, sensory, and motor deficits. Radiological imaging (MRI or CT angiography, Doppler ultrasound or angiography) should be reviewed. The occurrence of bilateral carotid artery disease should be noted. Preoperative evaluation needs to emphasize the neurological system but also the cardiac system, as the risk of myocardial ischemia is similar to the risk of neurological injury postoperatively [58, 59]. The history, physical examination, and investigations employed should focus on the identification of angina, myocardial infarction, congestive cardiac failure and the presence of arrhythmias. Hypertension is a treatable and preventable risk factor for the development of stroke as the incidence of postoperative neurological deficits is greater in patients with uncontrolled preoperative hypertension [59]. McCroy et al. reported in a multicenter trial that the diastolic pressure of >110 mmHg was a predictor of adverse outcome [60]. Hence there may be reason to delay elective surgery if the preoperative blood pressure is greater than 180/110 mmHg [61]. The question of the extent of cardiac investigations in each patient is debatable and one should use established guidelines [62]. Other risk factors include diabetes, hypercholesterolemia, peripheral vascular disease and chronic obstructive pulmonary disease [63]. Cigarette smoking has been identified as a risk factor for carotid restenosis [8]. Control of blood glucose levels is needed, as there may be a negative relationship between plasma glucose and outcome after stroke [64, 65], however data suggests that surgery can be performed safely in patients with diabetes [65, 66]. History of cervical arthritis and appearance of TIA symptoms with certain head and neck positions should be noted, to avoid precipitating cerebral ischemia during intraoperative head positioning. Most patients with carotid artery stenosis will be on some form of antiplatelet therapy or anti coagulation. Though these drugs may impair coagulation, there is not a consensus as when or if they should be stopped prior to surgery. The Cochrane review concluded that antiplatelet drugs can reduce the risks of stroke in patients undergoing CEA, while they may increase the risk of hemorrhage [67]. There were too few data to quantify the effect, and hence the recommendation supported the routine use of antiplatelet drugs in patients having CEA [67]. This decision needs to be made in conjunction with the neurosurgeon or neurologist.

## 9. Extracranial-Intracranial Bypass Surgery

Extracranial-intracranial (ECIC) bypass surgery is performed for cerebral revascularization in patients with history of cerebral ischemia such as patients with cerebral vascular occlusive disease and moyamoya, and for replacement of blood flow where sacrifice of a vessel is required during complex aneurysm or tumor surgery [68, 69]. The preoperative assessment requires careful review of their neurological system as many will present with TIA or stroke. Comorbidities may include other systemic disorders that are associated with moyamoya such as sickle cell disease. Intraoperative factors that may aggravate cerebral ischemia include hypotension, hypoxemia, hypovolemia, anemia, and hyperglycemia. The patient should be optimized to prevent these. Blood pressure manipulation is frequently required especially during temporary occlusion of a cerebral artery, thus preoperative blood pressure assessment and documentation is critical. 

## 10. Interventional Neuroradiology

The endovascular treatment of many neurosurgical patients includes the coiling and stenting of aneurysms, embolisation of AVM, arteriovenous fistula, and vascular tumors, and stenting of carotid artery stenosis [70]. The anesthetic considerations in this group of patients include maintenance of physiological stability, manipulation of systemic and regional blood flow, managing anticoagulation, and associated complications. Assessment and preparation of these patients also involves ensuring that they are suitable for radiological procedures. Pregnancy should be excluded in the child bearing age group. Renal function should be noted as contrast medium employed during imaging can worsen preexisting renal insufficiency [71]. Documentation of patients' history of adverse reaction to contrast dyes requires pretreatment with steroids and anti histamines [72]. Coagulation status of the patient as well as a history of allergy to protamine sulphate needs documentation as anticoagulation is employed during these procedures. As the radiology suite may be remote from the main operating rooms any possible difficulties during anesthesia should be noted to allow for preparation by the anesthesiologist. This includes patients with significant or unstable cardiac and respiratory disease, a difficult airway or a patient who requires additional monitoring not routinely available in the radiology suite. A careful airway assessment should include attention to the history of snoring as the resultant movement artifact distorts the quality of the images when the patient is awake or sedated. Most endovascular procedures can be performed with general anesthesia or conscious sedation techniques. Frequently general anesthesia is preferred to ensure an immobile patient for good imaging and less chance of rupture of vessels or lesion. Discussion of the anesthetic management with the patient needs to include the technique of anesthesia and if awake they need reassurance and information about what to expect during the procedure. 

Endovascular therapy with the use of coils and stents is an accepted mode of treatment of many cerebral aneurysms, though there is controversy [73]. The preoperative concerns and preparations are the same as for the surgical treatment of the aneurysms. If endovascular treatment is not successful many of these patients will then undergo surgery. Embolization of an AVM is performed as a complete treatment modality especially when the AVM is not suitable for surgical resection due to difficult anatomical access or location. A preoperative embolization may be performed for large AVM to minimize intraoperative blood loss. Attention to the preoperative base line blood pressure is important as manipulation of hemodynamics includes induced hypotension and even the use of adenosine to stop the heart during delivery of the embolic materials. Carotid artery stenting is a reasonable alternative, to carotid endarterectomy, particularly in patients at high-risk [74]. These patients may have difficult surgical anatomy, but more concerning to the anesthesiologist are the medical co morbidities. The preoperative assessment in the high risk patient will require a multidisciplinary approach to optimize areas of physiological concerns such as blood pressure, heart rate, oxygenation, and hemoglobin. Most frequently these procedures are performed with conscious sedation and careful monitoring of the cardiac and neurological systems [75]. Documentation of the baseline heart rate during the preoperative assessment is important as severe bradycardia and asystole can be anticipated during this procedure, especially during balloon inflation. Patients with elevated systolic blood pressures are at an increase risk of hemodynamic instability and neurological events during stent application [76]. 

## 11. Functional Neurosurgery: Deep Brain Stimulators

Functional neurosurgery with the use of deep brain stimulators (DBS) began and was initially successful in patients with Parkinson's disease. Its applications have since expanded to treat disorders such as dystonia, tremors, epilepsy, and chronic pain. The preferred technique of anesthetic management in many centers is local anesthesia with monitored care [77]. However both conscious sedation and general anesthesia are also used. Meticulous preoperative assessment is needed to optimize this population of often elderly patients with multiple comorbidities. The patients should be assessed for their ability to cooperate and ability to tolerate the various stages of this surgery which include placement of a head frame, preoperative imaging, and the insertion and testing of the DBS. As MRI is often performed prior to the DBS lead implantation, meticulous history of pacemakers, aneurysm clips or implanted ferrous metal should be obtained. Contraindication to DBS includes patient non compliance, dementia, medical co morbidities that preclude safe surgery, and extensive brain atrophy. Assessment of the airway is critical especially for the awake patients as a fixed head frame, which is secured to the operating room table is used, making emergent manipulation of the airway difficult [78]. Preoperative preparation includes instruction and reassurance of the patient for the requirements of procedures. Medications used to treat motor symptoms are often withheld on the morning of the surgery. Premedications such as opiods, benzodiazepines, and other sedatives can interfere with the interpretation of tremor and hence is avoided. The decision to preoperatively withhold anti Parkinson drugs may result in a patient with severe symptoms of rigidity or tremors. 

Patients with Parkinson's disease are often elderly with a classic triad of resting tremor, muscle rigidity, and bradykinesia with loss of postural reflexes [79] and multisystem problems (Table 4). There are also the potential drug interactions and adverse reactions from anti-Parkinson's medications (Table 5) [78].

## 12. Epilepsy Surgery

Patients with intractable epilepsy not controlled with medical therapy are candidates for surgical resection of their seizure foci [80]. A number of different procedures requiring anesthesia are performed during the investigations and treatment. This includes insertion of cortical electrodes for mapping and activation of the epileptogenic focus, and craniotomy for resection of the focus that may be with awake craniotomy or under general anesthesia, with or without electrocorticography and stimulation testing [81]. Communication among all team members, including neurologist, surgeon, and anesthesiologist, is vital to the successful preparation of these patients for the chosen technique of anesthesia. The administration of anticonvulsant agents prior to surgery is done in consultation with the neurologist and surgeon. Specific concerns of patients with epilepsy include the accompanying medical problems such as psychiatric disorders, neurofibromatosis, and multiple endocrine adenomatosis. Specific anesthetic considerations include adverse effects of antiepileptic drugs such as confusion, sedation, ataxia and nausea and vomiting. Most anticonvulsants are metabolized in the liver and lead to induction of liver enzymes that increase the metabolism of other drugs, particularly anesthetics. Other side effects from drugs include depresses the hemopoietic system and cardiac toxicity. Long-term treatment with phenytoin causes gingival hyperplasia with poor dentition and potentially difficult airway management. 

## 13. Summary

Anesthesia for the patient undergoing a neurosurgical and endovascular procedure presents special considerations. The brain is a highly vascular organ enclosed within a rigid skull. Tolerance of the brain to disruption of substrate delivery is minimal. Anesthetic and physiological parameters controlled by the anesthesiologist have profound effects on the cerebral homeostasis. The appropriate preoperative evaluation and preparation of the patient is critical to the success of the outcome. An understanding of the pathophysiological disturbances, the systemic manifestations associated with the disease state, the procedure and its special requirements form the essence of a through preoperative assessment. Communication with the patient and family, as well as the neurosurgeon and neuroradiologist, will ensure a good understanding of the anticipated events and allow for a well-prepared patient.

## Figures and Tables

**Table 1:** Glasgow Coma Scale.

Parameter	Score
*Eye opening*	
Spontaneously	4
To speech	3
To pain	2
None	1

*Best verbal response*	
Orientated	5
Confused	4
Inappropriate words	3
Incomprehensible sounds	2
None	1

*Best motor response*	
Obeys commands	6
Localizing pain	5
Withdraws from pain	4
Flexion to pain	3
Extension to pain	2
None	1

Patients with a scale score less than 7 require intubation for airway protection.

**Table 2:** Pituitary tumors and frequency of occurrence.

Pituitary tumor	Hormone secreted	Frequency of occurrence
Prolactinoma	Prolactin	20–30%
Cushing's disease	Adenocorticotropic hormone	10–15%
Acromegaly	Growth Hormone	5–10%
Gonadotroph	Follicle stimulating Hormone/Lutenising hormone	5%
Thyrotropic	Thyroid Stimulating Hormone	<3%
Mixed cell adenoma	None	20–30%
Nonfunctioning adenomas	None	20%

**Table 3:** Differences between diabetes insipidus and syndrome of inappropriate antidiuretic hormone.

	Diabetes insipidus	Syndrome of inappropriate
		antidiuretic hormone
Presentation	Polyuria Thirst (awake patients)	Hyponateriamia
		
Serum sodium	Increased >145 mEq/L	Decreased <135 mEq/L
		
Serum osmolarity	High >310 mOsm/L	Low/hypotonic <275 mOsm/L
		
Urine volume	Increased volume >4 L/day	Low
		
Treatment	Supportive fluid replacement and DDAVP	Fluid restriction with hypertonic sodium correction if serum sodium <120 mEq/L

**Table 4:** Systemic effects of Parkinson's disease.

System	Clinical signs/symptoms	Relevant investigations
Central nervous system	Muscle rigidity, Tremors, Depression, Hallucination, Akinesia	Neurological assessment Psychological assessment
Autonomic nervous system	Difficulty or alteration in salivation, Gastrointestinal function, Temperature regulation, Seborrhoea	Gastrointestinal studies
Cardiovascular system	Hypertension, Arrhythmias, Orthostatic hypotension	Electrocardiogram, Echocardiogram
Respiratory system	Retained secretions, Atelectasis, Respiratory Infections, Aspiration Pneumonia	Chest radiograph, Arterial blood gases, Spirometry, Pulmonary function test
Gastrointestinal system	Dysphagia, Sialorrhoea, Esophageal dysfunction, Weight loss	Serum albumin, electrolytes, creatinine
Endocrine system	Altered glucose metabolism	Blood glucose level

**Table 5:** Medical therapy for Parkinson's disease.

Drugs	Side effects
Dopamine agonist	
Bromocriptine, Carbegoline,	Nausea/vomiting,
Ramipraxole, Ropinirole	Delusions/hallucinations,
	Hypotension, Dysrhythmias

Dopamine precursors	
Levodopa, Carbidopa, Sinemet	Similar to Dopamine Agonist

Anticholinergics	
Benzotropine	Confusion/hallucination,
	Sedation/drowsiness, Dry mouth,
	Urinary retention

MAOI-B inhibitors	
Selegiline	Diarrhea
